# OIE/FAO International Scientific Conference on Avian Influenza

**DOI:** 10.3201/eid1211.061013

**Published:** 2006-11

**Authors:** Nina Marano

**Affiliations:** * Centers for Disease Control and Prevention, Atlanta, Georgia, USA

**Keywords:** OIE/FAO, Scientific Conference on Avian Influenza, Avian flu, bird flu, Alejandro Schudel, Michel Lombard,

Since 1997 the international community has witnessed a strain of highly pathogenic H5N1 avian influenza virus emerge and spread at an unprecedented rate. It has had devastating consequences for domestic poultry, wild avian species, and humans on 3 continents; 240 human cases have occurred with 141 deaths. From 1999 to 2003, poultry outbreak control measures in the European Union alone resulted in the depopulation of 50 million birds at a substantial cost to the global economy. Because of the ongoing human and animal infections, the public health and veterinary communities have recognized the urgent need for an ongoing collaborative and participatory approach to prevention and control of highly pathogenic avian influenza (HPAI).

This monograph contains the proceedings of the International Scientific Conference on Avian Influenza held in Paris, France, in April 2005. The conference was sponsored by the World Organization for Animal Health (OIE) and the Food and Agriculture Organization (FAO), in collaboration with the World Health Organization (WHO). To address the emerging animal health crisis and mitigate risks to human health, at the outset of the HPAI H5N1 outbreaks in Southeast Asia in early 2004, these organizations held joint meetings in Rome (February 2004), followed by 2 regional meetings in Bangkok (February 2004) and Ho Chi Minh City (February 2005) to issue guidelines and recommendations for prevention and control. Because it appears that this HPAI H5N1 epizootic will persist for some time, the Paris 2005 meeting was held to achieve consensus on the most current strategies for long-term prevention and control, including poultry vaccination when appropriate.

Bernard Vallat, the director general of the OIE, opened the meeting and urged scientists and regulators to consider strengthening farm biosecurity measures; to assess the role of ducks as a reservoir for avian influenza; to evaluate animal vaccination strategies; and to promote strengthening of veterinary services to enable better detection, surveillance, and response as an "international public good." This meeting marked the official launching of the OIE/FAO Network (OFFLU), a network of avian influenza reference laboratories created to promote research on avian influenza, provide technical assistance to developing countries on diagnosis and management, and serve as a mechanism to interface with the WHO Influenza Network to obtain virus isolates from animals that can be used to produce vaccines to prevent a human pandemic.

Opening remarks were made by Ilaria Capua, director of OIE and the National Reference Laboratory for Newcastle Disease and Avian Influenza in Padua, Italy. Dr. Capua called on the veterinary scientific community to take the following actions to limit the spread of the outbreaks: 1) expand understanding of the role of waterfowl and other nongallinaceous birds in the ecoepidemiology of HPAI, 2) further define the role of poultry vaccination in reducing the spread of infection and promoting animal welfare, 3) educate workers about prevention of exposure to avian influenza, and 4) conduct studies to address food safety concerns.

More than 300 internationally renowned scientists with expertise in avian influenza attended the meeting, which featured sessions on ecology and epidemiology, pathogenesis, human health implications, diagnostics, control strategies including vaccination, and improvement of management tools. Highlights of the scientific recommendations generated include an emphasis on global sharing of viral isolates, research on epidemiology of wild birds, research on mechanisms of transfer between wild and domestic avian species, and research on pathogenesis in other farmed birds to clarify their role as intermediate hosts. The scientists concluded that the following elements were critical for achieving long-term control of HPAI infections in animals and humans: monitoring viruses for antigenic changes in virulence, performing surveillance of H9N2 viruses with the potential to infect mammals, and conducting epidemiologic studies at the human-animal interface by the OIE/FAO and the WHO networks of reference laboratories.

This monograph contains the full text of the introductory speeches and manuscripts upon which the invited talks and abstracts of the poster sessions were based. It is an excellent reference for anyone interested in understanding the challenges the public health and veterinary community are facing due to the rapid emergence and complex ecoepidemiology of a viral pathogen that represents a major threat to public health and animal well-being.

